# HOPE: Hybrid-Drive Combining Optogenetics, Pharmacology and Electrophysiology

**DOI:** 10.3389/fncir.2018.00041

**Published:** 2018-05-16

**Authors:** Sebastien Delcasso, Sachira Denagamage, Zelie Britton, Ann M. Graybiel

**Affiliations:** Department of Brain and Cognitive Sciences, McGovern Institute for Brain Research, Massachusetts Institute of Technology, Cambridge, MA, United States

**Keywords:** electrophysiology, optogenetics, drug delivery, microdrive, multi-site, electrode array

## Abstract

Understanding the neural mechanisms underlying human cognition and determining the causal factors for the development of brain pathologies are among the greatest challenges for society. Electrophysiological recordings offer remarkable observations of brain activity as they provide highly precise representations of information coding in both temporal and spatial domains. With the development of genetic tools over the last decades, mice have been a key model organism in neuroscience. However, conducting chronic *in vivo* electrophysiology in awake, behaving mice remains technically challenging, and this difficulty prevents many research teams from acquiring critical recordings in their mouse models. Behavioral training, implant fabrication, brain surgery, data acquisition and data analysis are all required steps that must be mastered in order to perform cutting-edge experiments in systems neuroscience. Here, we present a new method that simplifies the construction of a drivable and multi-task electrophysiological recording implant without loss of flexibility and recording power. The hybrid-drive combining optogenetics, pharmacology and electrophysiology (HOPE) can support up to 16 tetrodes, attached to a single drive mechanism, organized in two bundles of eight tetrodes, allowing recordings in two different mouse brain regions simultaneously with two optical fibers for optogenetic manipulation or two injection cannulas for drug-delivery experiments. Because it can be printed with a latest-generation desktop 3D printer, the production cost is low compared to classical electrophysiology implants, and it can be built within a few hours. The HOPE implant is also reconfigurable to specific needs as it has been created in a computer-aided design (CAD) software and all the files used for its construction are open-source^1^.

## Introduction

Single-unit recordings made from tetrodes have been useful in linking neuronal activity to animal behavior. In particular, these methods allow detection of circuit dynamics if applied to populations of neurons at different nodes of neural circuits. *In vivo* electrophysiological recordings in awake, behaving mice have been particularly powerful for validating and studying different models of neuronal disease (Cacucci et al., 2008; Cayzac et al., 2011; Burguière et al., 2013). Unfortunately, the fabrication of neural implants for such recordings in mice is time-consuming, and commercially available implants are expensive. In order to facilitate the fabrication of neural implants and reduce their cost of production, we have created a new type of implant and a set of useful construction tools. The development of stereolithography 3D printing gives researchers the opportunity to build compact, versatile and easily modifiable implants. The use of this technology requires the editing of a very precise plan for each part, which can be easily done via the graphic interface of a computer program. As the implant then could be rapidly printed for a low cost, it could be modified as necessary to meet the needs of a specific experiment. We have made such a system.

Intracerebral drug delivery enables external regulation of neurotransmitter pathways and has been successful in identifying neurotransmitter functions as well as in counterbalancing pathological neurotransmitter levels. The combination of electrophysiological recordings and intracerebral drug-delivery systems enables neuroscientists to study the impact of neurotransmission levels on brain activity at levels ranging from neural networks to single cells. Such combinations can also be used in closed-loop systems to adjust drug dosage according to the recorded neural response. These approaches are still very challenging (Kliem and Wichmann, 2004) and therefore extremely rare in clinical treatment of humans or in non-human primate research. Rodent research plays a key role in the development of these new closed-loop drug delivery systems.

The development of optogenetic tools has provided the ability to manipulate specific neuronal networks and pathways at an extremely fine timescale (Boyden et al., 2005; Zhang et al., 2006). The combination of optogenetic manipulation with electrophysiological recordings adds a new dimension to electrophysiological recordings by allowing the user to be certain of the recorded cell-type (Friedman et al., 2015a, 2017). One can know whether the recorded cell is a projection neuron or an interneuron and even what input-output connections it has (Wickersham et al., 2010).

Here, we have developed a method to permit use of these electrophysiological, pharmacologic and optogenetic methods in flexible combinations. Particular values of our system are ease of manufacture and low initial cost.

## Materials and Methods

### Implant and Construction Tools

**Figure 1:**
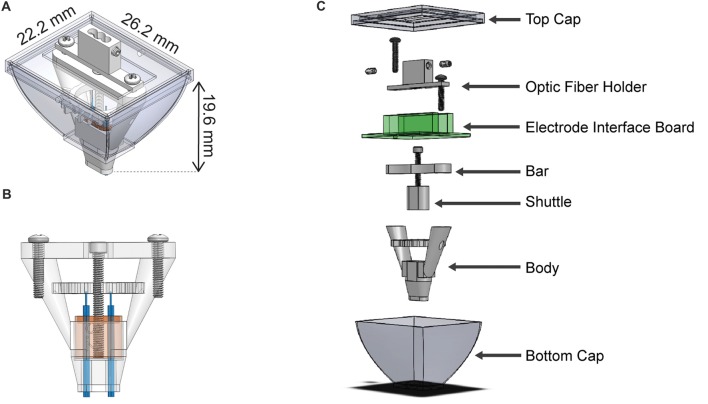
Schematic representation of the hybrid-drive combining optogenetics, pharmacology and electrophysiology (HOPE). **(A)** Outer view with dimensions. **(B)** Section view showing the drive mechanism used to move the shuttle. **(C)** Exploded view of the implant with all individual parts.

**Figure 2:**
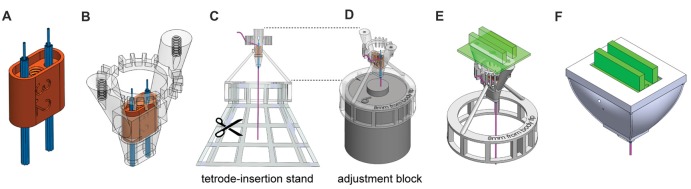
Steps of HOPE-implant construction. **(A)** Insertion of the two polyimide-tube bundles into the shuttle. **(B)** Insertion of the shuttle into the body. **(C)** Insertion and cutting of the tetrodes. **(D)** Leveling of tetrode lengths. **(E)** Addition of the drive system (screw and bar) and the EIB. **(F)** Encapsulation of the implant.

We designed the HOPE implant to incorporate a 64-channel electrode interface board (EIB, Neuralynx, Bozeman, MT, USA) that is 26.2 mm wide, 22.2 mm long and 19.6 mm high (Figure 1A). The height of the implant results from the presence of a drive mechanism (Figure 1B), allowing all of the electrodes to move up and down over a distance of 4 mm. The weight of the implant is 4.4 g, and its production cost is extremely small (<2 US$). Six 3D printed parts, an EIB and five screws (Figure 1C) need to be assembled in order to build the implant. The central piece to which all the others are connected is called the body. The second part, called the shuttle, carries the electrodes and is penetrated by a 0–80 screw at its center. As the shuttle fits snugly into the body, a full rotation of this 0–80 screw will generate a vertical movement of 317.5 μm, moving the electrodes up and down. The third part, called the bar, holds the same screw against the EIB and prevents the screw from moving vertically. The fourth part, called optic fiber holder, is only present when the implant also carries optical fibers for optogenetic experiments. Two set screws are used for locking the fibers in place when they need to be connected to a patch cord, preventing them from breaking when a strong stress is applied to them. The fibers will be unlocked when the shuttle needs to move. Finally, the fifth and sixth pieces are the top and the bottom caps, which protect the implant from contamination or damage when the animal is in its home cage. In addition to the implant, three useful construction tools were designed to help in the fabrication of the implant. The first is called the tetrode-insertion stand, which serves to hold the body during the insertion of the tetrodes (Figure 2C). The second is the adjustment block, which is used to level the tetrodes (Figure 2D). Finally, the third tool is a storage box to prevent damage to the implant when it has been fabricated. Each piece has been created using a 3D computer-aided design (CAD) software (Solidworks, Dassault Systems, France).

### Fabrication of the Implant

The designed pieces were 3D-printed using a Form2 printer (Formlabs, Cambridge, MA, USA). The printed parts were cleaned, and support material was carefully removed. The central channel in the shuttle was tapped using a 0–80 tap. The size of the shuttle was decreased manually, with sandpaper, until it fit perfectly into the body. The tightness of the fit increases the stability of the recordings by minimizing possible movement of the shuttle. Two bundles of nine polyimide tubes (12 mm long, 0.0062″/0.0092″ ID/OD, HPC Medical Products) were then assembled and inserted on each side of the shuttle (Figure 2A). After insertion, the polyimide tubes protruded from the bottom of the shuttle by 5.5 mm. The bundles were glued to the top of the shuttle, which has a rim that holds the glue, using a light cure adhesive (Loctite 4305). The part of the bundle extruding from the bottom of the shuttle was also covered with a thin layer of glue to hold all the polyimide tubes together, being careful to avoid sealing the polyimide-tube openings.

The alignment of the tetrode bundle is directly related to the alignment of the polyimide-tube bundle; this step has to be executed very carefully. After attaching both bundles to the shuttle, the shuttle was inserted into the body (Figure 2B). The drive mechanism (bar, screw, shuttle and body) presented in Figure 1B was tested at this point, and a top-view diagram of polyimide-tube bundles was drawn. The body was then mounted on the tetrode-insertion stand (Figure 2C), and the tetrodes were inserted into the polyimide tubes one by one. Tetrodes were made following the standard fabrication protocol (Jog et al., 2002; Nguyen et al., 2009), and the dimension of the wire and the number of turns required to create the tetrode varies depending of the desired final length of the tetrode as well as the diameter of the original wire used to build it. After insertion, the upper part of the tetrode was bent to fit within the teeth of the body. The ring of teeth located at the top of the body separated the tetrodes based on their connection to the EIB above. The tetrodes extended 1.5 cm above the top of the ring. The original top-view diagram was updated after the insertion of each tetrode so that one knew their location relative to the bundle of polyimide tubes. This information on the location of the tetrode is used in later analysis for histological assessment of tetrode position. After tetrode insertion, the stand was removed, and with fine scissors, each bundle was cut at the level of the ring located under the body. For final leveling of the tips of the tetrodes, the adjustment block was inserted into the body bottom-ring, and the tetrode tips were leveled. The tetrodes were then glued to the polyimide tubes on the top side of the shuttle. The adjustment block (Figure 2D) was then removed, and the drive mechanism (bar and screw) was assembled. The EIB was then mounted on the body, and tetrode wires were connected to it (Figure 2E). Using a nanoZ (Neuralynx, Bozeman, MT, USA) and a gold solution, the electrodes were gold-plated to lower their impedance to 200–300 kΩ (Jog et al., 2002). After gold plating, the electrodes were rinsed using deionized water. Finally, the protective bottom-cap and a top-cap were mounted and glued in place (Figure 2F). These fabrication procedures are described in detail in the Supplementary Presentation.

### Animals and Surgical Procedures

In this series of experiments, 15 C57BL/6 mice (Jackson Laboratory, Bar Harbor, ME, USA) and four parvalbumin (PV)-IRES-Cre (B6;129P2-*Pvalb^tm1(cre)Arbr^*/J) × Ai39 (B6;129S-*Gt(ROSA)26Sor^tm39(CAG-hop/EYFP)Hze^*/J) mice (generated from PV-Cre and Ai39 lines purchased from Jackson Laboratory) were studied. All experimental procedures were in accordance with the Guide for the Care and Use of Laboratory Animals of the National Research Council and were approved by the Committee on Animal Care at the Massachusetts Institute of Technology, Cambridge, MA, USA. For all microdrive implantations, we followed a precise series of surgical steps. Each mouse was deeply anesthetized with isoflurane (3% isoflurane in oxygen at 0.8 l/min flow rate), the head was shaved, and the mouse was head-fixed in a stereotaxic frame. The eyes were protected with ointment, the head was centered by adjusting ear-bar positions, the skin was cleaned three times with Betadine followed by 70% alcohol, and an incision was made in the skin to expose the calvarium. A sterile plastic ring was inserted underneath the skin to maintain the opening, the exposed region was cleaned, and the skull was leveled using the dorsoventral coordinates of Bregma and lambda. Two shallow openings were made in the skull with a 0.7 mm drill (AP = +4.0 mm, ML = ±2.5 mm). The drill was stopped before the skull was fully perforated. Two skull screws were firmly attached to the skull at these two sites. Two craniotomies were initiated at AP = 0.85 mm and ML = ±1.75 mm using a large circular drill bit (1.5 mm diameter). A small surgical hook was used to complete the craniotomy opening. The dura mater was incised using a small 30G needle bent near the tip, the dura mater was removed with fine forceps, and then the drive, attached to a stereotaxic arm, was lowered. The ground wire was wrapped around the skull screws, and the connection was strengthened using a drop of conductive silver paint on top of the head of the screw. Once the silver paint had dried, the screw was covered with a drop of ultraviolet (UV) curing glue (Loctite Flash Cure 4311 Cyanoacrylate), and the connection was sealed with 10 s of UV light. The drive was then lowered extremely slowly into the brain until the tetrodes reached the desired dorsoventral coordinates (−2.5 mm). Finally, the HOPE drive was attached to the skull and was anchored to two skull screws using Metabond quick self-curing cement.

### Data Acquisition and Analysis

All neuronal signals were band-pass filtered between 0.1 Hz and 9000 Hz and recorded at 30 kHz using Open Ephys^2^. Spikes were extracted offline from continuous data using custom Matlab scripts. Spike sorting was performed with custom Matlab programs (Friedman et al., 2015b). The results of the automatic spike-sorting algorithm were opened in Offline Sorter to correct misclassifications of units and inclusion of noisy spikes by the automatic clustering algorithm. In such cases, the experimenter redrew manually the borders of the cluster. The projection of the peak values of all spikes as well as the average waveforms of each cluster were exported directly from Offline Sorter. Peri-event time histograms were generated using custom Matlab scripts.

### Optogenetic Manipulations

For optogenetic experiments, an optical fiber (100 μm diameter, Doric Lenses, Quebec City, Canada) was placed at the center of each bundle. The distance from the tip of each tetrode to the tip of the optic fiber was adjusted by hand to 500 μm. The efficiency of light transmission was measured experimentally before implantation, and the power of the laser (593-nm light-pulses, 500 ms duration, randomly separated by 5.5–8 s) was adjusted to 5 mW during stimulation experiments.

### Drug Delivery

For pharmacological experiments, one guide cannula was attached to the center of each bundle and adjusted such that the guide tips were located 1 mm above the tips of the tetrodes. During these experiments, one injection cannula was inserted into each guide cannula so that the tip of the injection cannula extended 500 μm beyond the end of the guide cannula. Muscimol was prepared in batches and stored at 4°C until 1 h before injection, when the muscimol was brought to room temperature. At the chosen site, 300 μl of muscimol was injected using an infusion pump (Stoelting Co., Wood Dale, IL, USA) to control precisely the volume and flow rate of the intracerebral injection.

## Results

### Recording Activity of Striatal Neurons

**Figure 3:**
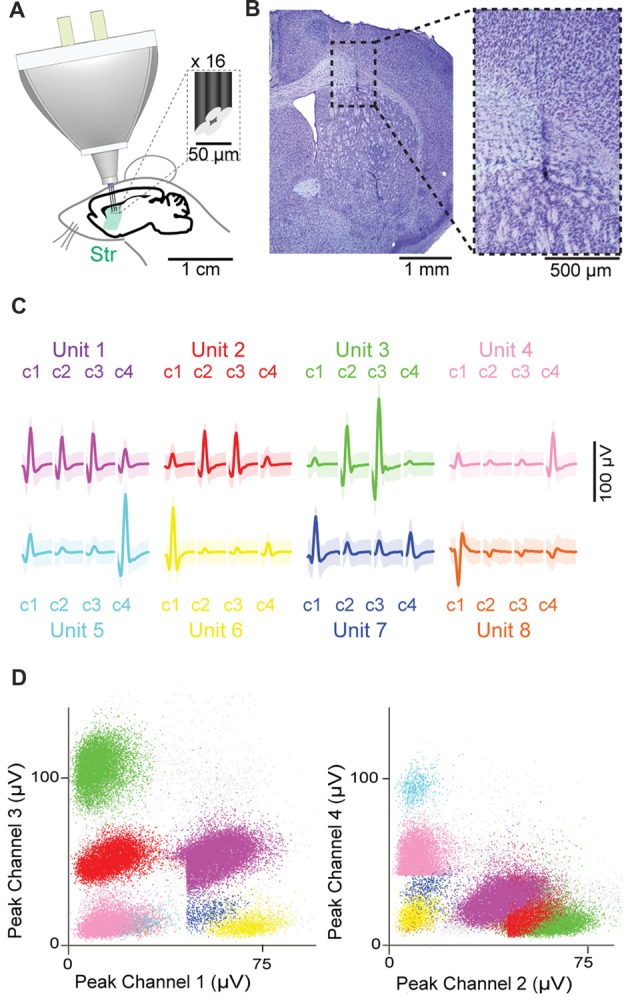
Chronic recordings in the dorsal striatum. **(A)** Schematic representation of the recording preparation. Inset shows the tip of a tetrode at high magnification. **(B)** Histological images showing the position of the tetrode tip in the striatum. **(C,D)** Eight isolated single units recorded by four channels (c1–c4) of a single tetrode, with waveforms of each single unit **(C)** and scatter dot plots based on the magnitude of their peaks on each channel **(D)**. Shading in **(C)** represents three standard deviations around the mean.

The HOPE device was implanted in 15 C57BL/6 mice, and 16 tetrodes divided into two bundles of eight tetrodes were targeted to contralateral districts within the dorsal striatum (Figure 3A). The locations of the recording sites were confirmed with histological analysis of Nissl-stained sections (Figure 3B). The signals originating from different neurons recorded with a single tetrode were classified into putative single units based on the peak value of the action potentials recorded on the four wires of the tetrodes (Figures 3C,D).

### Optogenetic Manipulation of Striatal Parvalbumin-Positive Interneurons

**Figure 4:**
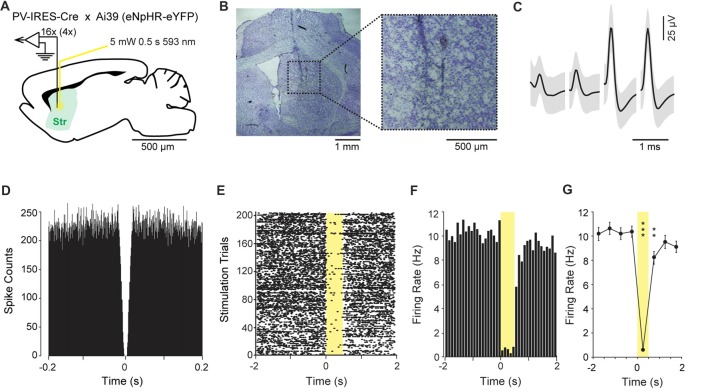
Optogenetic inhibition of parvalbumin (PV)-positive neurons in the striatum. **(A)** Schematic representation of the recording and light delivery location in the striatum (Str). **(B)** Histological confirmation showing the position of the tetrodes. **(C)** Average waveform of one isolated single unit recorded on the four channels of one tetrode. Shading indicates three standard deviations around the mean. **(D)** Autocorrelogram of the unit shown in **(C)**, confirming that there is no spike during the firing refractory period. **(E)** Raster plot showing the activity of the unit shown in **(C)**, during ±2 s around the illumination onset. Yellow shading shows the period of light delivery. **(F)** Peri-stimulus time histogram showing reduction of the firing rate during illumination. **(G)** Average firing rate during consecutive 500-ms intervals around light delivery. Error bars represent one standard deviation around the mean. **P < 0.01, ***P < 0.001.

In a second experiment, we optogenetically identified striatal neurons expressing PV. For this purpose, the HOPE device was upgraded with two optic fibers and then implanted in four PV-IRES-Cre × Ai39 mice (Figure 4A). The locations of the tetrodes as well as the locations of the optic fibers were confirmed by post mortem histology to be in the dorsomedial striatum (Figure 4B). In the striatum, the estimated proportion of PV-positive interneurons is small (<5%). The average waveform of an optogenetically identified neuron and the autocorrelogram of its action potential timestamps are shown in Figures 4C,D. The zero spike counts near the center of the autocorrelogram (time 0), corresponding to the refractory period of the unit, indicate that only one unit was isolated in this cluster.

During the experiment, the firing rate of this neuron systematically dropped to almost zero during each of the 205 illuminations of yellow light (593 nm, 5 mV, 500 ms duration; Figure 4F). The peri-event time histogram, which represents the average firing rate around the stimulations, shows that the firing rate of this unit decreased by *ca.* 10-fold when the yellow light was applied through the optic fiber (Figures 4E,F). The effect of the illuminations was analyzed by multiple *t*-test comparisons between 500 ms time-periods (Figure 4G). The analysis focused on a 4-s time-period centered at the onset of the illumination. The first 500 ms time bin was used as the baseline. We did not observe significant variation in firing rate preceding the onset of the illumination (*t*_(204)_ = −0.38, *P* = 0.70). The first significant difference was observed when the light was turned on, and we observed a sharp decrease in the firing rate during the 500-ms period with light delivery (*t*_(204)_ = 18.04, *P* < 0.0001). The firing rate increased slowly but was still statistically below the baseline level 500 ms after the light was turned off (*t*_(204)_ = 3.07, *P* < 0.001). The level of activity was fully recovered 1 s after the light was turned off (*t*_(204)_ = 0.97, *P* = 0.33).

### Pharmacological Manipulation of Striatal Neurons

**Figure 5:**
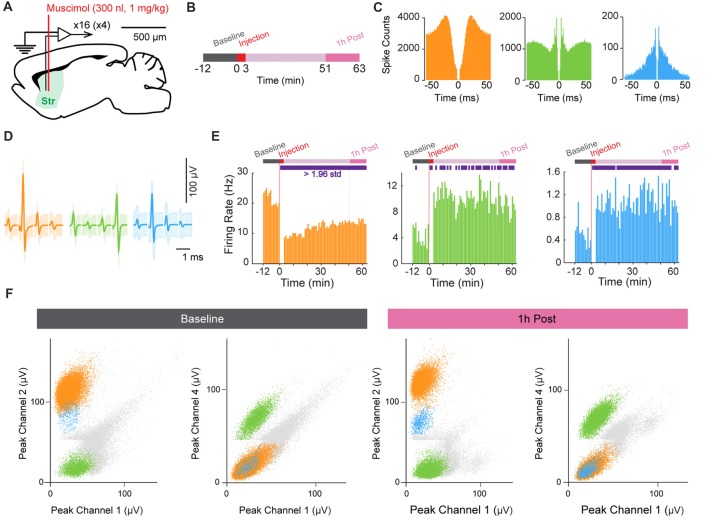
Muscimol injection in the striatum. **(A)** Schematic representation of the recording and injection location in the striatum (Str). **(B)** Experimental design. **(C,D)** Autocorrelograms **(C)** and average spike waveforms **(D)** of isolated single units simultaneously recorded from one tetrode. Shading in **(D)** indicates three standard deviations around the mean. **(E)** Firing-rate histograms showing the time course of the changes in firing rate before and after muscimol injection (*t* = 0). Purple rectangles in the schematic experimental design (top) indicate the period when the signal was significantly different from the baseline period (>1.96 standard deviation away from the mean). **(F)** Scatter dot plots of single units simultaneously recorded during the baseline period before muscimol injection (left) and 1-h post-injection period (right). Spikes are plotted based on the peak amplitude of the waveforms recorded on the four channels of the same tetrode.

In a third experiment, we injected muscimol (1 mg/kg) into the striatum while recording electrophysiological activity using the HOPE drive in a head-fixed mouse preparation (Figure 5A). The acquisition of neural data started 12 min before the beginning of drug injection (Figure 5B). Muscimol (0.3 μl) was injected over 3 min at a flow rate of 0.1 μl/min, and neuronal activity was not recorded during this period because the electric micro-pump generated electrical noise. Following the injection period, neuronal data were collected for 1 h. The waveform shapes of units recorded during the last 12 min of this recording period were used for direct comparison with those recorded during the baseline period to test the stability of the recorded units after injection of the muscimol (Figure 5F). Figures 5C,D shows the autocorrelograms and average waveforms of three isolated units recorded on the same tetrode and exhibiting modulation of their firing rates after muscimol injection. The injection of muscimol induced a decrease of firing rate in one unit (orange), whereas the two other units (green and blue) increased their firing rates following injection (Figure 5E). Finally, we confirmed the stability of recording by comparing cluster distributions for units recorded during the pre-muscimol baseline period and those recorded during the period at the end of the experiment (Figure 5F).

## Discussion

We present the design of a novel neural implant that we show can be used to record and to manipulate neuronal activity. Compared to previously developed implants (Battaglia et al., 2009; Nguyen et al., 2009; Voigts et al., 2013; Brunetti et al., 2014; Liang et al., 2017), the main characteristic of the presented implant is that it can be built easily by beginners, and it carries 16 movable tetrodes along with two optic fibers or two injection cannulas. Moreover, the HOPE implant has been designed with the help of 3D CAD software, allowing modifications for various experimental needs, such as targeting different brain regions and use in other animal species (e.g., rats and non-human primates). The implant can be designed to the shape of the skull of individual monkeys based on pre-implantation magnetic resonance imaging, thus optimizing the fit with the skull surface and making the recording from deep brain regions more reliable. As the different parts of the implant are 3D printed, it becomes inexpensive to produce the implant with an affordable desktop 3D printer. In fact, the price of the 3D printer is the price of one traditional commercially available implant for rats, and the implants themselves cost ~2 US$ in printing materials.

Despite these advantages, we should note that there are limitations to the HOPE implant. The weight of the implant could be an issue for young mice. This limitation could be overcome by making the body hollow inside and printing it with titanium to increase its strength. Also, during fabrication of the implant, the current design requires many steps to make individual parts. Cutting edge hybrid 3D printers can already produce a print made of different materials, such as plastic and silver in a single print. In the near future, it may be possible to print the entire implant in a single run, and multiple fabrication processes may become unnecessary.

The current HOPE implant is equipped only with a single drive to move all 16 tetrodes. However, with the hybrid 3D printing technology, the fabrication of implants with individual microdrives for each of these tetrodes may become possible. Such independently movable tetrodes are necessary when the target region consists of a thin layer of neurons. When larger regions are targeted, a simple drive mechanism could have a good yield, especially considering that adjusting each tetrode independently requires the experimenter to monitor and to isolate daily the units recorded from each tetrode. If few or no units are present, the experimenter generally decides to move the tetrode to identify more units. In order to move the tetrode, the animal must be slowly habituated to the procedure, as the experimenter will gently hold its head to make a small adjustment of the tetrode tip position by turning the screw of the tetrode carrier. At that time, there is a small risk that the tetrode will be disconnected from the circuit board if the animal tries to escape, or if the screwdriver slips on the head of the screw. One can wait for hours after lowering multiple tetrodes individually to make sure that the distance between the neurons and the tetrode is fully stabilized. With our design, we can still make adjustment of the tetrode location by moving all tetrodes at once if the quality of the recordings is getting too low or if the implants need to be removed from the brain. In some instances, this strategy can be advantageous for electrophysiological studies, where the number of animals is usually too low, although we fully realize the advantages of independent movement of the tetrodes during the course of prolonged experiments in mice (Kubota et al., 2009) and larger rodents (Jog et al., 1999; Barnes et al., 2005; Thorn et al., 2010; Delcasso et al., 2014).

In sum, the flexibility of the HOPE drive presented here suggests that as new methods are introduced, this drive could be adapted for efficient use. With currently available methods, this device is effective and can be supported at low cost, and thus it is available to many interested in recording the activity of neurons in the brain as animals engage in a range of behaviors.

## Author Contributions

SDelcasso created the HOPE design. SDelcasso and AG designed the experimental tests of the HOPE. SDelcasso, SDenagamage and ZB fabricated and tested the HOPE. SDelcasso analyzed the data with input from AG. SDelcasso and AG wrote the manuscript.

## Conflict of Interest Statement

The authors declare that the research was conducted in the absence of any commercial or financial relationships that could be construed as a potential conflict of interest.

## Footnotes

^2^www.open-ephys.org
