# Supplementary material for: HOPE: Hybrid-Drive Combining Optogenetics, Pharmacology and Electrophysiology
Source: Front Neural Circuits. 2018 May 16;12:41. doi: 10.3389/fncir.2018.00041 (PMC5972628; doi:10.3389/fncir.2018.00041)
Supplement: Supplementary file 1 [file Presentation_1.PDF]

FABRICATION OF THE HOPE DRIVE

https://delcasso.github.io/hope/

TETRODE FABRICATION

1. Wash your hands.
2. Cut a 30 cm section of 20 µm diameter tungsten wire using serrated scissors.
3. Fold the wire in half and run your fingers along the length of the wire to stick the strands together. Deionized water can be applied if necessary to keep the strands together.
4. Cut the extremity of the loop.
5. Fold the wire in half again to form a loop on one end and cut the tips of the wires on the other end to ensure that they are aligned.
6. Clip the four ends together using an alligator clip that has been modified with a horizontal bar and weights (Figure 1).
7. Hang the wire directly above a turning device and place the bar of the alligator clip into the tetrode spinner (Figure 2).
8. Apply 115 clockwise turns followed by 15 counterclockwise turns.
9. Melt the insulation of the wires to fuse them together. Use a heat gun at the highest temperature setting (1000°F) to heat the wire from four different directions.
10. For each direction, move the heat gun back and forth along the length of the wire twice at a distance of 1-2 cm, spending approximately 4 seconds on each pass.

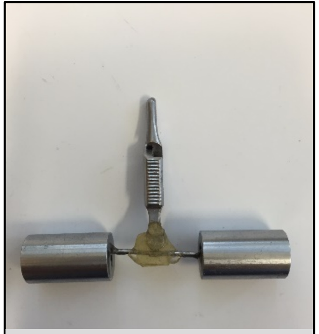

FIGURE 1

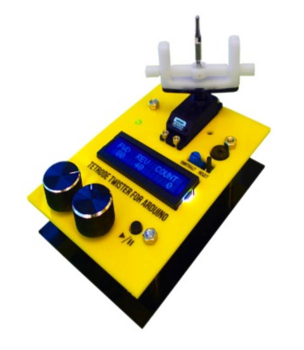

FIGURE 2

11. Lift the alligator clip slightly to release the tension from the weights and cut the tetrode above the tip of the alligator clip. Transfer the new tetrode into an anti-static box for storage.

You can consult the following JoVE link for a video that describe the procedures for tetrode construction.
https://www.jove.com/video/1098/micro-drive-array-for-chronic-in-vivo-recording-tetrode-assembly

SHUTTLE ASSEMBLY

1. Remove all parts from the 3D printer (Figure 3)

2. Remove supports from 3D printed parts (Figures 4 and 5).

3. The shuttle needs to slide smoothly and should be tight inside the body. The shuttle can be sanded down once on each side to ensure a proper fit.

4. Also check that the tetrode adjustment block fit fully within the ring of the body (Figure 6).

5. Use an 0-80 tap to form threads along:
   a. the central hole of the shuttle (Figure 7)
   b. the two EIB posts on the body
   c. the two holes in the side of the body's shuttle chamber

   When you tap the hole, it is not good to try to go all the way in. When it becomes too difficult to turn, it is better to unscrew the tapping tool and blow in the hole.

6. Cut eighteen sections of M-tube (0.0062"/0.0092" ID/OD), each 12 mm in length, with a razor blade.

7. Insert 9 of these segments into both the left and right holes of the shuttle. If you plan to use optical fibers, then cut two more sections of M-tube, each 15 mm in length. Place on long M-tube in the center of each bundle (Figure 8). Optic fibers will be glued inside the middle tube when the bundle will be assembled. If you plan to perform drug injection, place an injection cannula at the center of the bundle. The injection cannula is usually located 500 µm above the tip of the tetrodes.

8. Adjust the position of the M-tubes until they protrude 5.5 mm from the bottom of the shuttle.

9. Apply drops of a light-curing adhesive around the M-tubes using a needle and cure with UV light (405 nm) for 10 seconds. Make sure to wear appropriate glasses to protect your eyes from UV light.

10. Ensure that no adhesive falls into the tapped central hole.
    a. *It may be easier to make one bundle first and then the other one.*

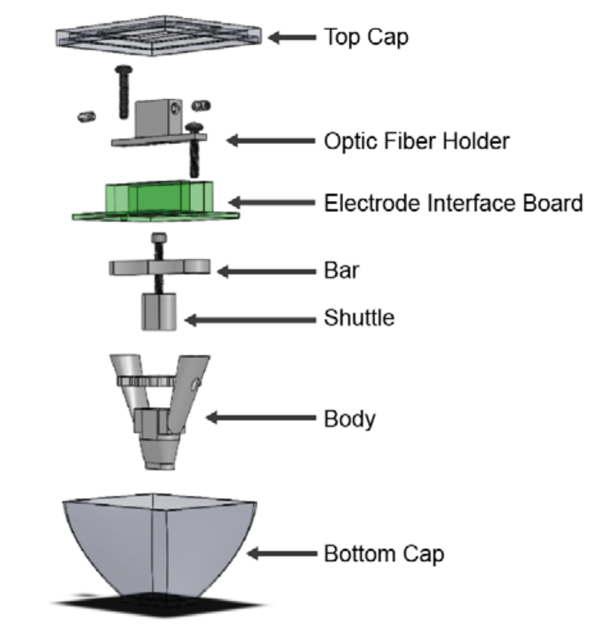

FIGURE 3

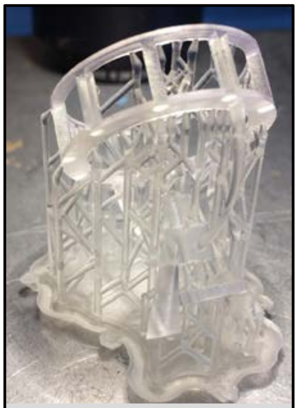

FIGURE 4

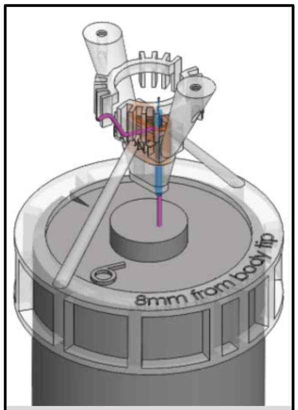

FIGURE 5

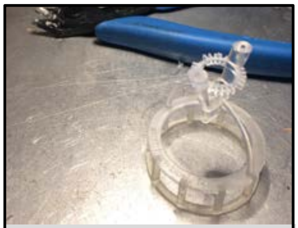

FIGURE 6

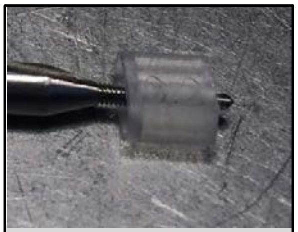

FIGURE 7

b. *Use a needle with epoxy and apply to M-tubes where they exit the base of the shuttle so that the epoxy travels by capillary action up into the shuttle hole and around M-tubes.*
c. *Apply down about ¾ the length of the M-tubes to ensure each M-tube is secured in the bundle.*

11. If necessary, adhesive can be applied slightly above the bottom of the M-tubes to hold them together in a tight cluster. Ensure that no adhesive enters through the bottom of the M-tubes and cure with UV light for 10 seconds. You can also use a second shuttle to hold the tip of the bundle when you are gluing it.

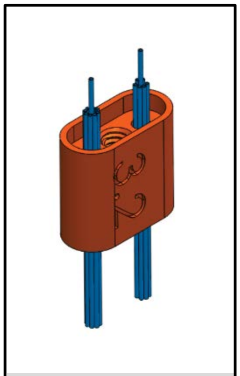

FIGURE 8

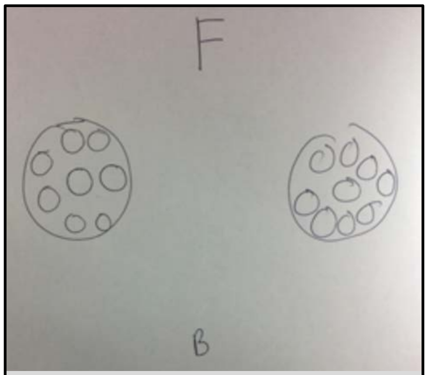

FIGURE 9

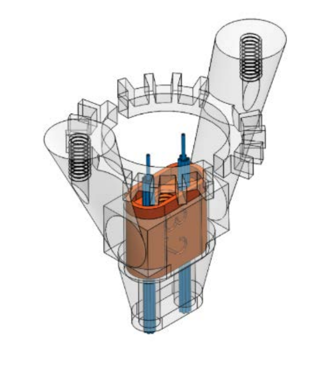

FIGURE 10

12. Draw exactly what you see (Figure 9) and label the front side with F (both on the diagram and on the shuttle itself)

13. Use the tap to guide the bottom of the shuttle into the body, making sure that the M-tubes slide through the holes in the drive body without bending. Make sure the shuttle is fully inserted into the body (Figure 10).

14. In preparation for loading tetrodes, with a fine needle (26G), test whether each M-tube is glued in place and does not slide down by touching lightly on the top of each. You can also use a needle to open any compressed M-tube openings.

TETRODE INSERTION

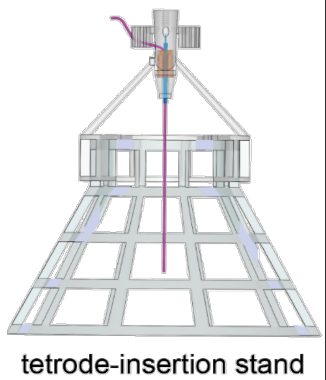

FIGURE 11

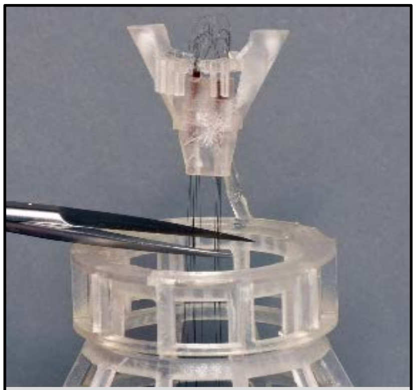

FIGURE 12

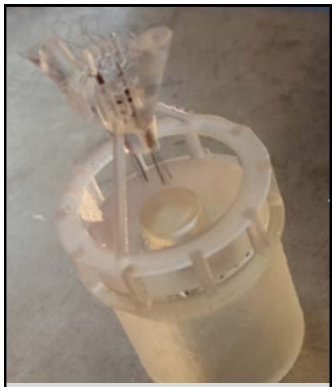

FIGURE 13

1. Place drive body into tetrode-insertion stand (Figure 11) and orient under scope. To aid proper orientation, drive frame should have a “Front” label.
    a. *Orient the “Front” label away from you, facing scope;*
    b. *With this orientation, tetrodes in the left bundle can be loaded and arranged left to right for attachment to EIBs #1 to 8. Right bundle can be loaded and arranged left to right on the bottom ring to attach to EIBs #16 to 9.*

2. Insert a tetrode into one of the M-tubes with a pair of forceps. Tap the frame so that the tetrode falls through M-tube. Gently grab the top of the loop at the end of the tetrode, pull up enough to pass it through one of the plastic gates and place it in an appropriate slot (for instance, to reach EIB pin holes #1-4) with ~1.5 cm slack from the slotted ring in order to have enough slack to eventually reach the EIB pins. *It is best performed under a microscope.*

3. Mark the position of the tetrode order on the diagram.

4. Repeat this process with all sixteen tetrodes and their corresponding plastic gates.

5. With a pair of serrated scissors, cut the tetrodes protruding from the bottom of the body in a single smooth and swift motion across the bottom of the shuttle body and the supporting cylindrical cage of the bottom (Figure 12).

6. Acquire a tetrode length adjuster (Figure 13), and lower the shuttle body and very gently tap the cut tetrode ends on the small cylinder in the tetrode length adjuster until they adjust to be even and flat.

7. Now that the tetrodes have been aligned, they must be secured to maintain their current position inside the M-tubes. Use a needle tip to carefully place a drop of the adhesive above the M-tubes and then cure with UV light for 10 seconds.

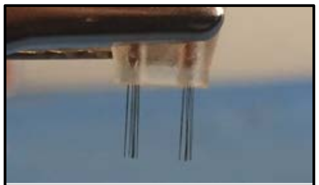

FIGURE 14

8. Important: Maintain awareness not to bend the tetrodes (Figure 14), and carefully remove the drive from the adjuster and replace it onto drive frame.

9. Make sure the shuttle has been fully pushed down.

10. Insert the screw into the bar, and then screw in the shuttle until the bar touches the body.

PREPARATION OF THE ELECTRODE INTERFACE BOARD (EIB)

1. Have an EIB ready.
2. Tap the screw holes on the EIB.
3. Align the bar on the bottom of the EIB using two 0-80 screws.
4. Make a small mark through the center hole of the bar onto the EIB.
5. Make a small hole with a hand drill and a 0.075” diameter drill bit.

MOUNTING THE ELECTRODE INTERFACE BOARD (EIB)

1. Place the electrode interface board onto the posts of the body and use 0-80 x 5/16” screws to secure it in place (Figure 15).
2. *Make sure to inspect the EIB and clear any extraneous epoxy from the back that might make the surface uneven; otherwise the printed bar may break.* There should not be any gap between the EIB and the bar or between the bar and the body. Tetrode 1 should face the front of the -body, and tetrode 16 should face the back.

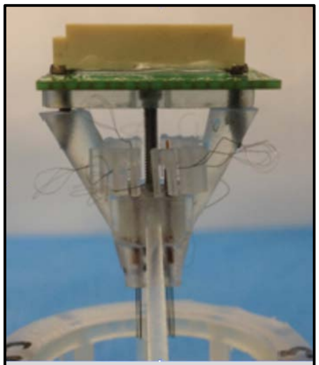

FIGURE 15

ELECTRODE INTERFACE BOARD (EIB)

1. Cut one of the tetrode loops with a pair of serrated scissors to produce four equal lengths of wire.
2. Forceps can be used to gently pull apart the wires if they are stuck together.
3. Each plastic slot on the body corresponds to one of the 16 sets of tetrode holes on the EIB board.
4. Thread the four separated wires of a tetrode through the four corresponding holes on the EIB board above with forceps and secure in place with gold pins.
5. Repeat this process with all sixteen tetrodes.

   Forceps quality makes a big difference. We use SPI PTFE-coated#5.
   Hold where the four wires meet to avoid bending near the ends (kinks make threading through EIB pin holes more difficult).

GROUND WIRE

1. Cut a 10 cm length of full hardness tungsten wire (0.005” Bare, 0.0070” Coated).
2. Use a torch to burn the insulation off the ends (burn 2 mm on each side).
3. Insert the fold through the ground wire hole (R2) in the EIB, with the longer end directed toward the far side of the device. The wire can be bent slightly so that it remains in place.

PROTECT THE DRIVE

1. Take the 3D printed cap and lower it onto the device. Secure in place by adding drops of light curing adhesive where the body meets the cap and curing with UV light for 10 seconds.

GOLD PLATING

1. Wash the tips of the tetrodes in baths of deionized water, ethanol, and then deionized water again to remove any residue.
2. Attach the fully assembled tetrode device onto the impedance testing apparatus and lower the tips of the electrodes into a bath of gold plating solution. The bath should be connected via a wire to the ground.
3. Open NanoZ on the connected computer and select “Test Impedances.” Initial values typically range from 200 to 400 kΩ.
4. Select “DC Electroplate” and switch to the “Match impedances” mode. The plating current should be set to −0.050 μA with a target of 200 kΩ at 1004 Hz, an interval of 1 second and a pause of 2 seconds. Choose an appropriate number of runs (if your impedances are higher, you will need more runs) and click on “autoplate.”
5. If the number of runs selected was not sufficient to bring the impedance below 200 kΩ, repeat the steps described above until the desired results are obtained. In cases where a tetrode was not connected properly or was constructed poorly, it may not be possible to lower the impedance significantly.

IMPLANTATION

1. Anesthetize the mouse with isoflurane (3% isoflurane in oxygen at 0.8 l/min flow-rate).
2. Shave the head.
3. Head-fix the mouse in a stereotaxic frame.
4. Protect the eyes with ointment.
5. Center the head by adjusting ear-bar positions.
6. Clean the skin three times with betadine followed by 70% alcohol.

7. Make an incision in the skin to expose the calvarium.

8. Insert a sterile plastic ring underneath the skin to maintain the opening.

9. Clean the exposed region.

10. Level the skull using the dorsoventral coordinates of bregma and lambda.

11. Make two shallow openings in the skull with a 0.7 mm drill (AP = +4.0 mm, ML = ±2.5 mm), and stop the drill before the skull was fully perforated.

12. Firmly attach two skull screws to the skull at these two sites.

13. Initiate two craniotomies at AP = 0.85 mm and ML = ±1.75 mm using a large circular drill bit (1.5 mm diameter).

14. Stop the drilling procedure when the skull became very thin.

15. Use a small surgical hook to complete the craniotomy opening.

16. Make an incision in the dura mater using a small 30G needle bent near the tip.

17. Remove the dura mater with fine forceps.

18. Lower the drive that is attached to a stereotaxic arm.

19. Wrap the ground wire around the skull screws, and strengthen the connection using a drop of conductive silver paint on top of the head of the screw.

20. Once the silver paint had dried, cover the screw with a drop of UV-curing glue (Loctite Flash Cure 4311 Cyanoacrylate), and seal the connection with 10 seconds of UV light.

21. Lower the drive extremely slowly into the brain until the tetrodes reached the desired dorsoventral coordinates (−2.5 mm).

22. Finally, attach the HOPE drive to the skull and the skull screws using Metabond quick self-curing cement (Figure 16).

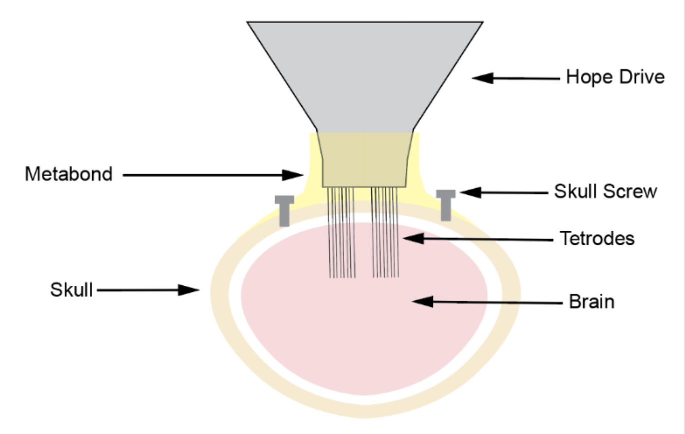

FIGURE 16

MATERIALS

- Loctite 4305 Light Cure Instant Adhesive (Henkel, IDH #303389)
- LED-200 UV Spot Curing System (Electro-lite, Part #81172)
- EIB-36-16TT (Neuralynx)
- 8975-6 11.6 AMP 570/1000-Degree Fahrenheit Dual Temperature Heat Gun (Milwaukee Tool Corporation)
- Tetrode Spinner (Neuralynx or open-ephys)
- 8 Micron Tungsten 99.95% CS Wire (California Fine Wire Company)
- Forceps *SPI PTFE-coated#5*
- 0.0062”/0.0092” ID/OD Medical Grade M-tubing (HPC Medical Products, IWG Lot # 1606000091)
- 3D printed cap, body and shuttle (Formlab Form2 + Clear Resine (GPCL02))
- Small gold plated electrode attachment pins (Neuralynx)
- 320 Grit Sandpaper (McMaster Carr, Part Number 4692A71)
- 0-80 General Purpose Tap (McMaster Carr, Part Number 2522A751)
- 0-80 x 1/8” Stainless Steel Pan Head Machine Screws (McMaster Carr, Part Number 91772A052) (attach EIB through bar to body)
- 0-80 thread size, ½” long, 18-8 stainless steel socket head (middle of the bar) screw (McMaster Carr, Part Number 92196A070).
- 26 GA injection cannula (Plastics One,C315G/SPC)
- 200 µm optic fibers (Thorlabs, CFMXB20)
- Skull screws 0.78 mm diameter (ANTRIN MINIATURE SPECIALTIES, INC.)
